# Physiological responses of coriander (*Coriandrum sativum* L.) to exogenous 2,4-epibrassinolide at different concentrations

**DOI:** 10.1186/s12870-023-04684-z

**Published:** 2023-12-16

**Authors:** Zhiqi Xu, Shuchao Huang, Yandong Xie, Shuya Wang, Ning Jin, Li Jin, Jianzhong Tie, Xin Meng, Zhaozhuang Li, Jian Lyu, Jihua Yu

**Affiliations:** 1 College of Horticulture, Gansu Agricultural University, Lanzhou, 730070 China; 2 State Key Laboratory of Aridland Crop Science, Gansu Agricultural University, Lanzhou, 730070 China

**Keywords:** EBR, Coriander, Photosynthetic parameters, Chlorophyll fluorescence, Growth and development, Quality

## Abstract

**Background:**

Brassinolide, known as the seventh plant hormone, can improve the photosynthetic capacity of plants, promote plant growth and development, promote the formation of horticultural crop yield, improve the quality of horticultural crops, and also improve the ability of plants to resist biological and abiotic stresses.

**Results:**

The effects of different concentrations of exogenously sprayed 2,4-epibrassinolide (EBR) on growth, physiological and photosynthetic characteristics of ‘All-round large leaf coriander’ were studied in substrate culture. The results showed that 0.05, 0.1, and 0.5 mg.L^− 1^ EBR promoted the growth of coriander and increased the aboveground fresh and dry weights, with 0.5 mg.L^− 1^ EBR having the most significant effect. Spraying 0.1 mg.L^− 1^ EBR increased the content of soluble sugars and protein of coriander leaves. Spraying 0.1 and 0.5 mg.L^− 1^ EBR significantly increased the chlorophyll content and photosynthetic parameters of coriander leaves, and 0.5 mg.L^− 1^ EBR also significantly increased the chlorophyll fluorescence parameters of coriander leaves. Spraying 0.5 mg.L^− 1^ EBR upregulated the expression of *CsRbcS*, *CsFBPase*, and *CsAld*. Correlation analysis showed that aboveground fresh weight under exogenous EBR treatment was significantly positively correlated with aboveground dry weight, plant height, *P*_*n*_, *G*_*s*_, *C*_*i*_, and *CsAld* (*P* < 0.05), and soluble sugar content was significantly positively correlated with the number of leaves, *Y*_(II)_, *q*P, and *CsRbcS*. The results of the principal component analysis (PCA) showed that there was a significant separation between the treatment and the control groups. Spraying 0.5 mg.L^− 1^ EBR can promote the growth of coriander, improve the quality of coriander leaves, and strengthen coriander leaf photosynthetic capacity. This study provides new insights into the promotion of coriander growth and development following the application of exogenous EBR.

**Conclusion:**

Exogenous EBR treatment increased coriander plant height, leaf growth and aboveground dry weight, and enhanced photosynthesis. Exogenous spraying of 0.5 mg.L^− 1^ EBR had the most significant effect.

**Supplementary Information:**

The online version contains supplementary material available at 10.1186/s12870-023-04684-z.

## Background

Coriander (*Coriandrum sativum* L.) belongs to the *Apiaceae* family, is a widely used plants with both medicinal and culinary properties [1]. Coriander is native to the Mediterranean region and cultivated in Asia, Central Europe, and North Africa [2]. The roots, stems, leaves, and seeds of coriander contain special fragrances. Coriander has a unique flavor and contains a variety of nutrients such as vitamins, mineral elements, and phenolic acid compounds, it also has antioxidant and anticancer effects [3, 4]. Coriander is used as an ingredient in the cuisines of China, Thailand, Vietnam, India, and other countries [5].

Brassinosteroids (BRs) are known as the sixth class of plant hormones. BRs play extensive roles in plant growth and development, the response to biological and abiotic stress, and optimizing plant photosynthesis, and affect both the yield of horticultural crops and the quality of horticultural products during postharvest storage [6]. To date, more than 70 brassinosteroids have been identified from variety of plants. Among them, brassinolide (BL), 2,4-epibrassinolide (2,4-EpiBL/EBR, EBR), and 28-homobrassinolide (28-HomoBL, HBL) show high biological activity [7]. EBR, a wildly available brassinosteroids, is a cost-effective plant hormone that affects plant growth and development at extremely low concentrations [8, 9]. Good growth conditions are required to improve the yield, quality, and health of coriander, but the possibility remains that judicious application of EBR might positively impact the growth of coriander.

Spraying with exogenous EBR can improve the yield of strawberries [10] and peas [11] and fresh and dry weight of Gladiolus (*Gladiolus grandiflorus* L.) [9]. It can boost the photosynthetic performance of horticultural crops such as pepper and has been shown to increase the activity ribulose-1,5-bisphosphate carboxylase/oxygenase (RuBisCO) in cucumber leaves, thereby improving the assimilation ability of CO_2_ in the Calvin cycle [12]. Dong’s [13] study showed that application of EBR could also increase the content of chlorophyll a (Chl a) and chlorophyll b (Chl b) in wheat (*T. aestivum* L. cv. Shannon 22) leaves. Stomata control the exchange of gas and water between plants and the external environment, and stomatal openness has a direct effect on the photosynthetic efficiency of plants, which forms the basis for dry matter formation [14]. Previous studies have shown that EBR can improve stomatal conductance (*G*_*s*_) of cucumbers (*Cucumis sativus*) [15]. Chlorophyll fluorescence is the endogenous light emitted by plants themselves, which is involved in energy distribution in plants together with photosynthesis. Photosynthesis is highly sensitive to environmental factors and different physiological states and environmental conditions can lead to differences in plant photosynthetic characteristics. Changes in the physiological characteristics of plants can be monitored using chlorophyll fluorescence [16]. Chlorophyll fluorescence is the light emitted by chlorophyll as it returns from excited to non-excited states, and it is widely used as a non-invasive monitor of photosynthesis. Previous studies have shown that exogenous EBR enhances the maximum quantum yield of PSII (*F*_*v*_*/F*_*m*_) and photochemical quenching (*q*P) in *Eucalyptus urophylla* [17] leaves. Yu et al. [12], showed that the initial activity of RuBisCO in cucumber leaves was significantly improved after 3 h of exogenous EBR treatment, however the total content and activity of RuBisCO in cucumber leaves were not significantly affected. Gao et al. [18], showed that exogenous EBR increased the activity of RuBisCO in maize protoplasts and also up-regulated the expression of dark-reaction enzymes glyceraldehyde-3-phosphate dehydrogenase subunit A (*GAPA*), cytoplasmic fructose-1,6-bisphosphatase (*cyFBPase*), ribulose-1,5-bisphosphate carboxylase small subunit (*RbcS*), phosphoenolpyruvate carboxylase (*PEPC*), fructose-1,6-bisphosphatase (*FBPase*), RuBisCO activase β subunit (*RCAβ*), ribulose-l,5-bisphosphate carboxylase large subunit (*RbcL*), *and* glutathione reductase (*GR*). Some studies have also shown that EBR can significantly increase the soluble sugar content of cucumber leaves and grapes (*Vitis vinifera*) [19], as well as the soluble protein content of olive (*Olea europaea*) [20] leaves. Studies on BRs have mainly focused on stresses, such as drought [21], low temperature [22], salt [23], and high temperature [24], and there have been few studies on the effects of exogenous spraying on the growth and physiology of vegetables. As a vegetable crop with both culinary and medicinal values, ‘All-round large leaf coriander’ was used as the test material in this study to investigate the effects of different concentrations of EBR spraying on the growth, yield per plant, quality, photosynthetic parameters, chlorophyll fluorescence parameters, and relative expression of genes encoding proteins important for carbon assimilation, The overall goal was to provide a theoretical basis for exogenous EBR spraying to promote the growth of coriander and to achieve yield improvement and quality enhancement as well as physiological response changes.

## Materials and methods

### Experimental materials and plant growth conditions

This study was conducted in the laboratory of the College of Horticulture, Gansu Agricultural University, starting on September 24, 2021. Seeds of ‘All-round large leaf coriander ' were produced from Gansu Anyan Agricultural Technology Co., Ltd. The EBR was purchased from Beijing Solarbio Technology Co., Ltd. The seeds were soaked for 6 hours and then put into a constant temperature (21℃) shaker for 48 h 100 RPM. The seeds were then sown into a 96-hole dish and put into an artificial climate chamber (RPN-400E-4; Ningbo Dongnan Instrument Co. Ltd., Zhejiang, China). The temperature of the artificial climate chamber was 20℃/17℃ (daytime/dark), photosynthetic photon flux density was 250 µmol m^− 2^s^− 1^, 12 h/12 h (daytime/dark). When the coriander seedlings had grown three true leaves, plants with uniform growth were selected and transplanted into plastic pots (7 × 7 × 8 cm) filled with a 3:1:1 (v/v/v) mixture of grass charcoal, vermiculite, and perlite substrate.

### Experimental design

This study used a completely randomized experimental design. Forty days after transplantation, coriander plants with uniform growth were selected for treatment. Five treatments were established with three replicates per treatment and six plants per replicate. Treatment settings were as follows: CK: water; T1:0.05 mg/L EBR; T2:0.1 mg/L EBR; T3:0.5 mg/L EBR; T4:1.0 mg/L EBR. All treatments involved foliar spraying, until both sides of all leaves showed moist condensation without dripping, once every three days, for a total of five times. One week after the treatment, the coriander reached the commodity standard, and the leaves were stored in a -80℃ refrigerator for index determination.

### Determination indices and methods

#### Morphological index determination

The height of the coriander plants was measured using a ruler, and the leaf area, circumference, length, and width were measured using a crop leaf morphology measuring instrument (TPYX-A, Zhejiang Top Cloud-Agri Technology Co.,Ltd.). The aboveground fresh weight of coriander was determined using an analytical balance. To measure the aboveground dry weight, the coriander was placed in a blast drying oven (HGZF-II/H-101-3, Shanghai Yuejin Medical Device Co., LTD) at 105℃ for 30 min, dried at 75℃ to constant weight, and then weighed using an analytical.

#### Determination of chlorophyll content

The chlorophyll content was determined according to the method described by Arnon [25]. A coriander leaf sample (0.1 g) was weighed and placed into a 25 ml stoppered test tube, to which was added 10 ml of 80% acetone. The tube was mixed thoroughly, sealed with plastic wrap, and chlorophyll was extracted for 48 h in the dark. The absorption values at 645 and 663 nm (A_645_ and A_663_) were determined using a UV-1800 visible spectrophotometer (SHIMADZU, Japan). Chl a, Chl b, and total chlorophyll content (Chl t) were calculated using the following formulas:

$$Chl\,a\,(mg.{g^{ - 1}}) = (12.71 \times A663 - 2.59\, \times A645) \times (V/1000 \times W)$$

$$Chl\,b\,(mg.{g^{ - 1}}) = (22.88 \times A645 - 4.67\, \times A663) \times (V/1000 \times W)$$

$$Chl\,t\,(mg.{g^{ - 1}}) = (20.29 \times A645 + 8.04\, \times A663) \times (V/1000 \times W)$$

where, *V* is the total volume of the extract and *W* is the fresh weight of the sample.

#### Determination of photosynthetic parameters

Net photosynthetic rate (*P*_*n*_), *G*_*s*_, transpiration rate (*T*_*r*_), and intercellular CO_2_ concentration (*C*_*i*_) were determined using a portable photosynthetic measurement system (CIRAS-2, PP-system, UK) from 9:00 to11:00 am on the fourth leaf from the bottom to the top.

#### Determination of chlorophyll fluorescence parameters

After placing the fourth leaf from bottom to top under dark conditions for 30 min, an IMAPING-PAM modulation fluorometer (Walz Germany) was used to determine the *F*_*v*_*/F*_*m*_, actual photosynthetic efficiency of PSII [*Y*_(II)_], non-photochemical quenching (NPQ), and *q*P.

#### Determination of quality indicators

Soluble protein content in coriander leaves was determined by the Kormas Brilliant Blue method [26]. The soluble sugar content of the coriander leaves was measured using an anthrone-sulfuric acid assay [27].

#### Real-time fluorescence quantitative polymerase chain reaction (RT-qPCR)

We searched for coriander carbon assimilation enzyme synthesis genes based on the annotations of key coriander carbon assimilation enzymes [28] and the coriander Genomics Database (CGDB, http://cgdb.bio2db.com/). The coding sequences for *CsFBPase*, *CsRbcL*, *CsRbcS* sedoheptulose-1,7-bisphosphatase (*CsSBPase*), triosephosphate isomerase (*TPiase*) and fructose-bisphosphate aldolase (*CsAld*) were downloaded from the CGDB, and the GenScript online website (https://www.genscript.com/) was used to design primers (Supplementary Table 1). A housekeeping gene (18s rRNA) [29, 30] was used as an internal reference, and the primers used were F: GTGGGCGATTTGTCTGGTT and R: TGTACAAAGGGCAGGGACGT. Total RNA extraction was performed according to the manufacturer’s instructions for the Accurate Biological Steady Pure universal RNA extraction kit (Hunan Accurate Biological Engineering Co., Ltd), and RT-qPCR reaction was performed according to the instructions of SYBR® Green *Pro Taq* HS premixed qPCR kit (Hunan Accurate Biological Engineering Co., Ltd). The RT-qPCR reaction system was 20 µL, comprising 2X SYBR Green *Pro Taq* HS Premix 10µL, Primer F 0.4 µL, Primer R 0.4 µL, RNase free water 8.2 µL. RT-qPCR reaction conditions: 95℃ 30 s, 95℃ denaturation 5 s, 60℃ annealing 30 s, 40 cycles. The relative gene expression was calculated by the 2^−ΔΔCT^ method.

### Statistical analysis

Excel 2016 was used to organize the data. The data were analyzed by one-way analysis of variance (ANOVA) using SPSS software (version 23.0; SPSS Institute Inc., Chicago, IL, USA), and significant differences were compared using Duncan’s multiple range test (*P* < 0.05). Correlation and principal component analyses (PCA) were performed using Origin 2022 (Origin Inc., San Francisco, CA, USA). Results are presented as mean ± standard error (SE).

## Results

### Effect of different concentrations of exogenous EBR on the growth of coriander

**Fig. 1:**
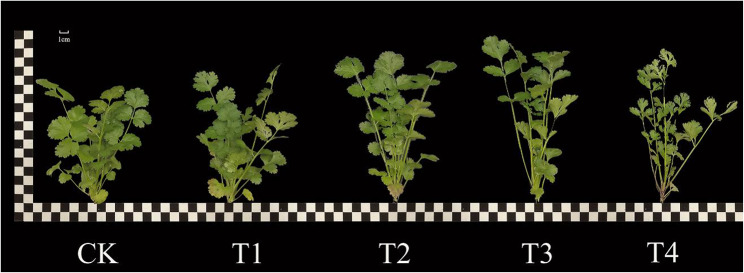
Effects of different concentrations of exogenous EBR on aboveground growth of coriander. CK: Water spraying; T1: 0.05 mg.L^− 1^ EBR; T2: 0.1 mg.L^− 1^ EBR; T3: 0.5 mg.L^− 1^ EBR; T4: 1.0 mg.L^− 1^ EBR

The number of leaves, leaf area, leaf circumference, leaf length, leaf width and plant height of coriander initially increased with an increase in the concentration of exogenous sprayed EBR concentration, and decreased at the highest concentration. Compared with CK (Table 1; Fig. 1), T3 significantly increased the number of coriander leaves, leaf area, leaf circumference, leaf length, leaf width, and plant height by 19.97%, 40.08%, 47.14%, 79.36%, 22.01%, and 17.45%, respectively. Compared with the CK, the T1, T2, and T4 treatments significantly increased the number of leaves and leaf length, the number of leaves increased by 11.40%, 14.22%, and 14.22%, respectively, and the leaf length increased by 33.97%, 37.68%, and 34.07%, respectively, and there was no significant difference in the number of leaves and leaf width between the T2 and T4 treatments. T4 treatment also significantly increased coriander plant height by 14.56%, T4 treatment significantly reduced the leaf area and leaf width of coriander, which were 16.94% and 5.32% lower than those of CK, respectively.

**Table 1:** Effects of different concentrations of exogenous EBR on the growth of coriander

Treatment	Number of leaves	Leaf area (cm^2^)	Leaf perimeter(cm)	Length of Leaf(cm)	Leaf width (cm)	Plant height (cm)
CK	11.67 ± 0.33^c^	25.80 ± 0.46^b^	46.78 ± 1.21^b^	9.98 ± 0.19^c^	49.24 ± 0.60^b^	14.90 ± 0.15^b^
T1	13.00 ± 0.00^b^	26.02 ± 0.36^b^	49.06 ± 1.96^b^	13.37 ± 0.30^b^	49.44 ± 0.84^b^	15.07 ± 0.27^b^
T2	13.33 ± 0.33^ab^	26.88 ± 0.38^b^	49.01 ± 0.75^b^	13.74 ± 0.20^b^	50.77 ± 0.29^b^	15.33 ± 0.33^b^
T3	14.00 ± 0.00^a^	36.14 ± 1.07^a^	68.83 ± 1.34^a^	17.90 ± 0.70^a^	60.08 ± 1.32^a^	17.50 ± 0.29^a^
T4	13.33 ± 0.33^ab^	21.43 ± 0.26^c^	51.13 ± 1.93^b^	13.38 ± 0.07^b^	46.62 ± 0.60^c^	17.07 ± 0.54^a^

Note: Values indicate mean ± SE (n = 3). Different lowercase letters in the columns represent significant differences among the treatments according to one-way ANOVA (Duncan’s test, *P* < 0.05). Abbreviations: CK: Water spraying; T1: 0.05 mg.L^− 1^ EBR; T2: 0.1 mg.L^− 1^ EBR; T3: 0.5 mg.L^− 1^ EBR;T4: 1.0 mg.L^− 1^ EBR

### Effects of different concentrations of exogenous EBR on aboveground fresh weight and aboveground dry weight of coriander

**Fig. 2:**
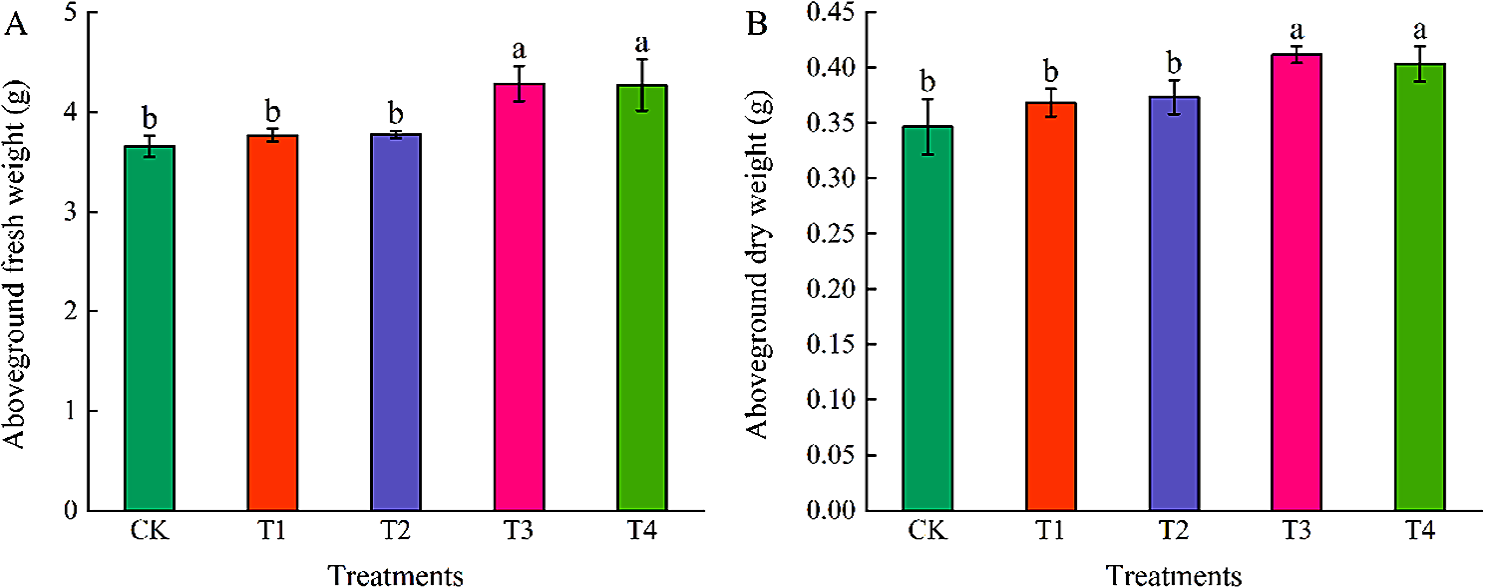
Effects of different concentrations of exogenous EBR on aboveground fresh weight **(A)** and aboveground dry weight **(B)** of coriander. CK: Water spraying; T1: 0.05 mg.L^− 1^ EBR; T2: 0.1 mg.L^− 1^ EBR,; T3: 0.5 mg.L^− 1^ EBR; T4: 1.0 mg.L^− 1^ EBR. Values presented are the means ± SE (*n* = 3). Vertical bars indicate the SE of the means. Different lowercase letters indicate significant differences between treatments (Duncan’s test, *P* < 0.05)

The aboveground fresh weight (Fig. 2A) and aboveground dry weight (Fig. 2B) of coriander first increased and then decreased with increase exogenous EBR concentration. Compared with CK, the aboveground fresh and dry weights of coriander increased under T1 and T2 treatments, but did not reach a significant level, T3 and T4 treatments significantly increased the aboveground fresh weight and aboveground dry weight of coriander, and the aboveground fresh weight and aboveground dry weight of T3 treatment increased by 17.24% and 18.75%, respectively, the aboveground fresh weight and aboveground dry weight of T4 treatment increased by 16.87% and 16.35%, respectively.

### Effects of different concentrations of exogenous EBR on chlorophyll content of coriander

**Fig. 3:**
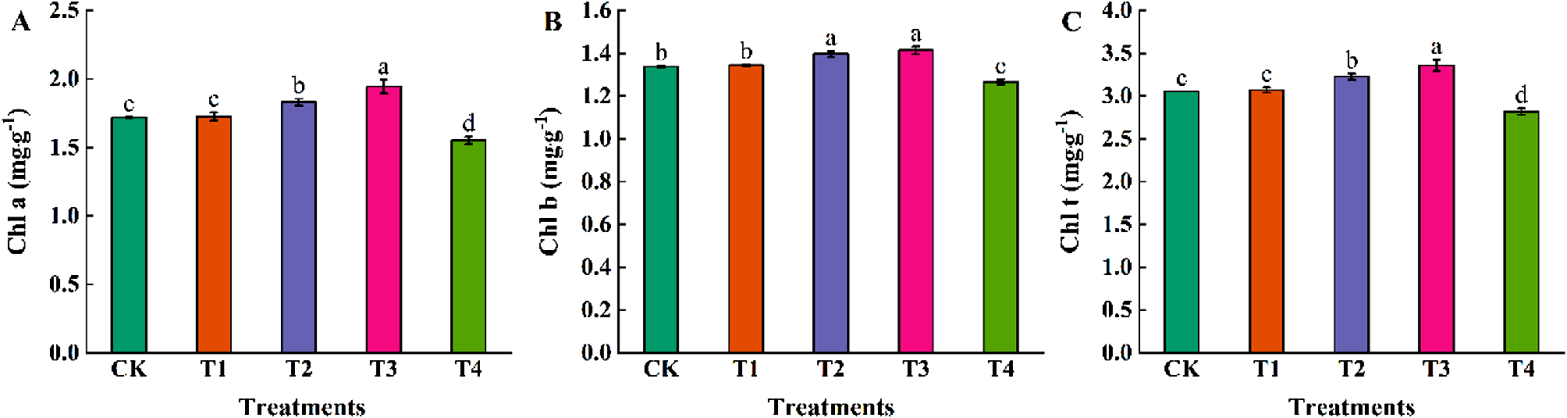
Effects of different concentrations of exogenous EBR on chlorophyll a **(A)**, chlorophyll b **(B)**, and chlorophyll t **(C)** content of coriander. Chl a: chlorophyll a, Chl b: chlorophyll b, Chl t: total chlorophyll. CK: water spraying; T1: 0.05 mg.L^−1^ EBR; T2: 0.1 mg.L^−1^ EBR; T3: 0.5 mg.L^−1^ EBR; T4: 1.0 mg.L^−1^ EBR. Values presented are the means ± SE (*n* = 3). Vertical bars indicate the SE of the means. Different lowercase letters indicate significant differences between treatments (Duncan’s test, *P* < 0.05)

To clarify the effect of EBR on the chlorophyll content of coriander, we determined the Chl a, Chl b, and Chl t contents in coriander leaves. The coriander Chl a (Fig. 3A), Chl b (Fig. 3B), and Chl t (Fig. 3C) contents showed an increasing trend and then decreased with an increase in exogenous EBR concentration. Compared with CK, T2 and T3 treatments significantly increased the contents of Chl a, Chl b, and Chl t in coriander leaves, and Chl a, Chl b and Chl t in coriander leaves under T2 treatment increased by 6.51%, 4.45%, and 5.61%, respectively; Chl a, Chl b, and Chl t in coriander leaves under T3 treatment increased by 13.18%, 5.76%, and 9.93%, respectively; and T4 treatment significantly reduced the content of Chl a, Chl b, and Chl t in coriander leaves. Chl a, Chl b, and Chl t increased in the T1 treatment, but the differences were no significant.

### Effects of different concentrations of exogenous EBR on photosynthetic parameters of coriander leaves

*P*_*n*_, *T*_*r*_, *G*_*s*_, and *C*_*i*_ of coriander leaves showed an increasing trend as exogenous EBR concentration increased and then decreased with a further increase in EBR concentration (Table 2). Compared with CK, T1-T4 treatments significantly increased *P*_*n*_, *T*_*r*_, and *G*_*s*_ of coriander leaves, and T2-T4 treatments significantly increased *C*_*i*_ of coriander leaves. The *P*_*n*_ of coriander leaves were increased by 44.21%, 63.19%, 97.91%, and 89.75% under T1-T4 treatments, respectively. *T*_*r*_ was increased by 40.28%, 55.48%, 81.27%, and 67.14% under T1-T4 treatments, respectively. *G*_*s*_ increased by 41.55%, 46.29%, 110.99%, and 109.21% under T1-T4 treatments, respectively. *C*_*i*_ of coriander leaf increased by 4.22%, 15.30%, and 9.32% under T2-T4 treatments respectively. There was no significant difference in *C*_*i*_ between the CK and T1 groups.

**Table 2:** Effects of different concentrations of exogenous EBR on photosynthetic parameters of coriander leaves

Treatment	*P*_*n*_ (µmol·m^− 2^·s^− 1^)	*T*_*r*_ (mmol·m^− 2^·s^− 1^)	*G*_*s*_ (mol·m^− 2^·s^− 1^)	*C*_*i*_ (µmol·mol^− 1^)
CK	5.27 ± 0.03^d^	2.83 ± 0.07^d^	112.33 ± 3.18^c^	379.00 ± 3.21^d^
T1	7.60 ± 0.26^c^	3.97 ± 0.09^c^	159.00 ± 2.89^b^	384.33 ± 2.96^d^
T2	8.60 ± 0.21^b^	4.40 ± 0.15^b^	164.33 ± 2.60^b^	395.00 ± 4.16^c^
T3	10.43 ± 0.26^a^	5.13 ± 0.12^a^	237.00 ± 3.79^a^	437.00 ± 2.89^a^
T4	10.00 ± 0.21^a^	4.73 ± 0.17^b^	235.00 ± 2.31^a^	414.33 ± 2.19^b^

Note: *P*_*n*_, net photosynthetic rate; *T*_*r*_, transpiration rate; *G*_*s*_, stomatal conductance; *C*_*i*_, intercellular CO_2_ concentration. CK: water spraying; T1: 0.05 mg.L^− 1^ EBR; T2: 0.1 mg.L^− 1^ EBR; T3: 0.5 mg.L^− 1^ EBR; T4: 1.0 mg.L^− 1^ EBR. Values indicate mean ± SE (*n* = 3). Different lowercase letters in the columns represent significant differences among different treatments according to one-way ANOVA (Duncan’s test, *P* < 0.05)

### Effects of different concentrations of exogenous EBR on the fluorescence parameters of coriander leaves

**Fig. 4:**
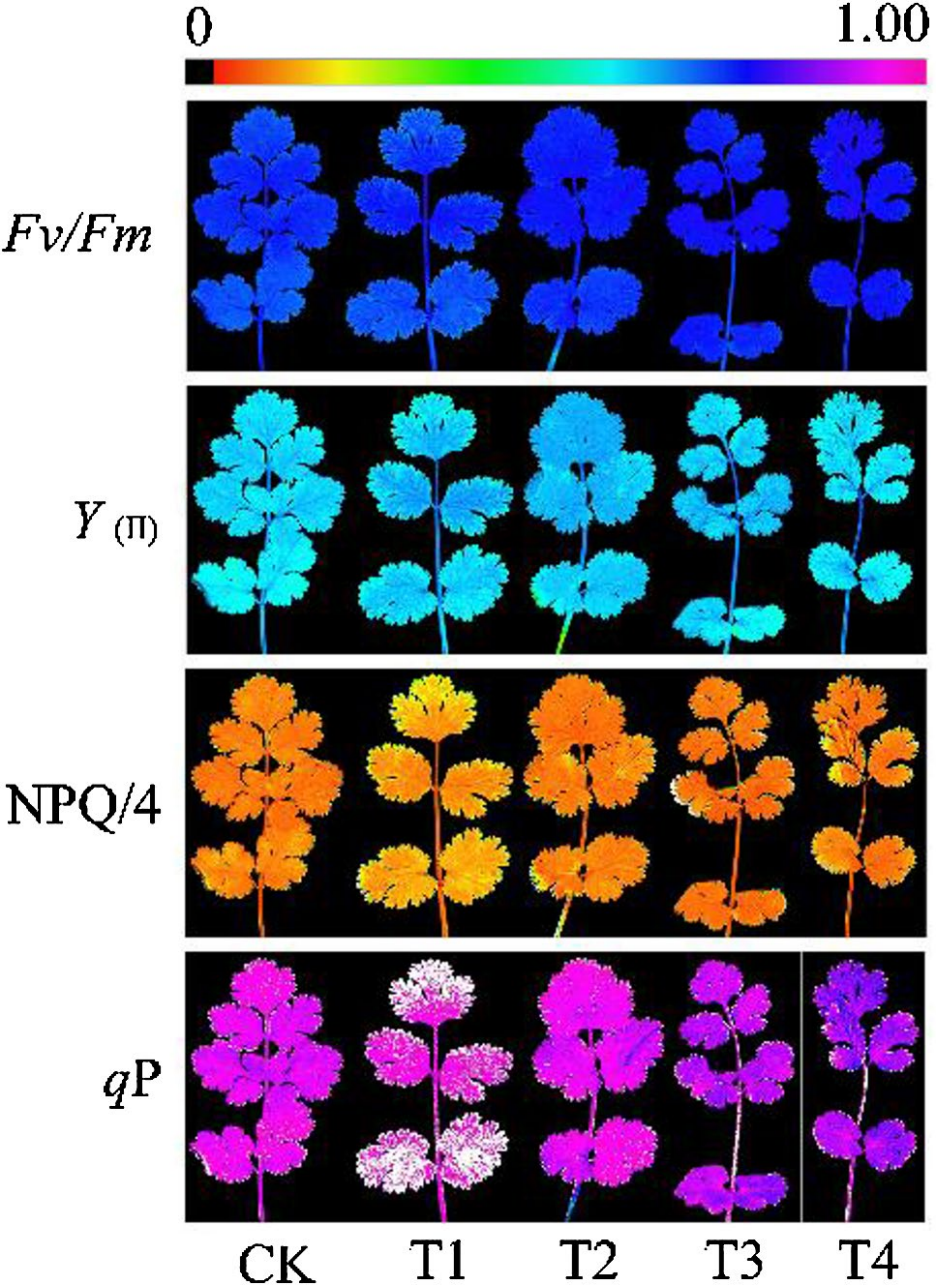
Effects of different concentrations of exogenous EBR on chlorophyll fluorescence imaging of coriander leaf. *Fv/Fm*: maximum photochemical efficiency of PSII; *Y*_(II)_: actual photosynthetic efficiency of PSII; NPQ: non-photochemical quenching; *q*P: photochemical quenching. CK: water spraying; T1: 0.05 mg.L^− 1^ EBR; T2: 0.1 mg.L^− 1^ EBR; T3: 0.5 mg.L^− 1^ EBR; T4: 1.0 mg.L^− 1^ EBR

To clarify the effects of exogenous EBR on PSII, we measured the chlorophyll fluorescence parameters. Under exogenous EBR treatment, *F*_*v*_*/F*_*m*_, *Y*_(II)_, NPQ, and *q*P of coriander leaves showed a trend of increasing and then decreasing with increasing EBR concentration (Table 3), which was consistent with the changes in *F*_*v*_*/F*_*m*_, *Y*_(II)_, NPQ/4, and *q*P fluorescence color (Fig. 4). Compared to CK, *F*_*v*_*/F*_*m*_ of coriander leaves increased by 1.69%, 2.62%, 3.52%, and 2.95% under the T1-T4 treatments, respectively; *q*P increased by 8.03%, 6.05%, 8.16%, and 4.68% under the T1-T4 treatments, respectively; *Y*_(II)_ of coriander leaves under the T3 treatment increased by 5.97%. The NPQ of coriander leaves under the T2 treatment increased by 17.78% compared to that of CK, whereas the NPQ of coriander leaves under the T1, T2, and T4 treatments did not differ significantly from that of CK.

**Table 3:** Effects of different concentrations of exogenous EBR on the fluorescence parameters of coriander leaves

Treatment	*F*_*v*_ */F*_*m*_	*Y*_(II)_	NPQ	*q*P
CK	0.7210 ± 0.0018^c^	0.5883 ± 0.0058^b^	0.1125 ± 0.0031^b^	0.8666 ± 0.0027^b^
T1	0.7332 ± 0.0038^b^	0.6016 ± 0.0079^ab^	0.1325 ± 0.0016^a^	0.9362 ± 0.0075^a^
T2	0.7399 ± 0.0034^ab^	0.6024 ± 0.0125^ab^	0.1198 ± 0.0069^ab^	0.9190 ± 0.0029^a^
T3	0.7464 ± 0.0027^a^	0.6234 ± 0.0009^a^	0.1148 ± 0.0059^b^	0.9373 ± 0.0119^a^
T4	0.7423 ± 0.0029^ab^	0.5894 ± 0.0074^b^	0.1073 ± 0.0041^b^	0.9072 ± 0.0195^a^

Note: *Fv/Fm*: maximum photochemical efficiency of PSII; *Y*_(II)_: actual photosynthetic efficiency of PSII; NPQ: non-photochemical quenching; *q*P: photochemical quenching. CK: Water spraying; T1: 0.05 mg.L^−1^ EBR; T2: 0.1 mg.L^−1^ EBR; T3: 0.5 mg.L^−1^ EBR; T4: 1.0 mg.L^−1^ EBR. Values indicate mean ± SE (*n* = 3). Different lowercase letters in the columns represent significant differences among different treatments according to one-way ANOVA (Duncan’s test, *P* < 0.05)

### Effects of different concentrations of exogenous EBR on soluble sugar and soluble protein in coriander leaves

**Fig. 5:**
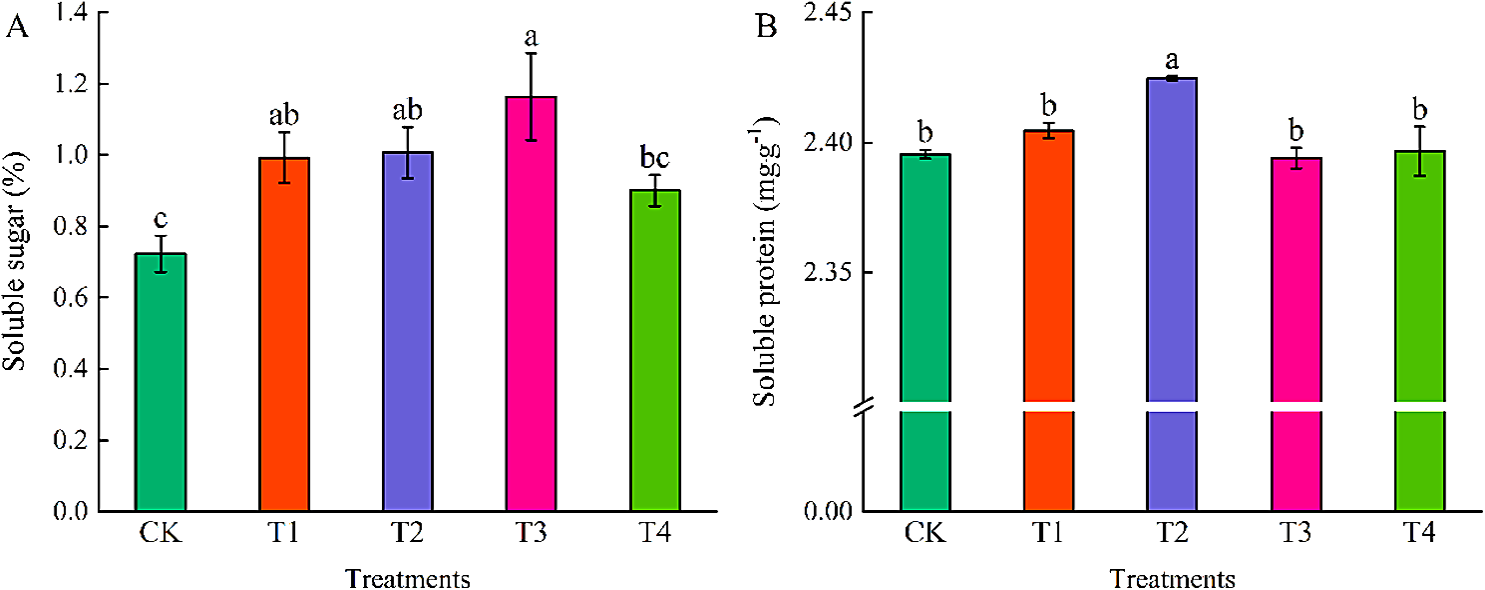
Effects of different concentrations of exogenous EBR on soluble sugar (A) and soluble protein (B) of coriander. CK: Water spraying; T1: 0.05 mg.L^− 1^ EBR; T2: 0.1 mg.L^− 1^ EBR; T3: 0.5 mg.L^− 1^ EBR; T4: 1.0 mg.L^− 1^ EBR. Values presented are the means ± SE (*n* = 3). Vertical bars indicate the SE of the means. Different lowercase letters indicate significant differences between treatments (Duncan’s test, *P* < 0.05)

Soluble sugars (Fig. 5A) and soluble proteins (Fig. 5B) of coriander first increased and then decreased with increasing exogenous EBR concentrations. Compared with CK, T1-T3 treatments significantly increased coriander soluble sugar content by 37.23%, 40.45%, and 60.93%, respectively, whereas the soluble sugar content of coriander leaves increased under T4 treatment, but the difference was not significant. Compared with the other treatments, the soluble protein content of coriander leaves in the T2 treatment was significantly increased and was 1.22%, 0.83%, 1.28%, and 1.33% higher than that in the CK, T1, T3, andT4 treatments, respectively.

### Effects of different concentrations of exogenous EBR on the expression levels of carbon assimilation key enzyme genes in coriander leaves

**Fig. 6:**
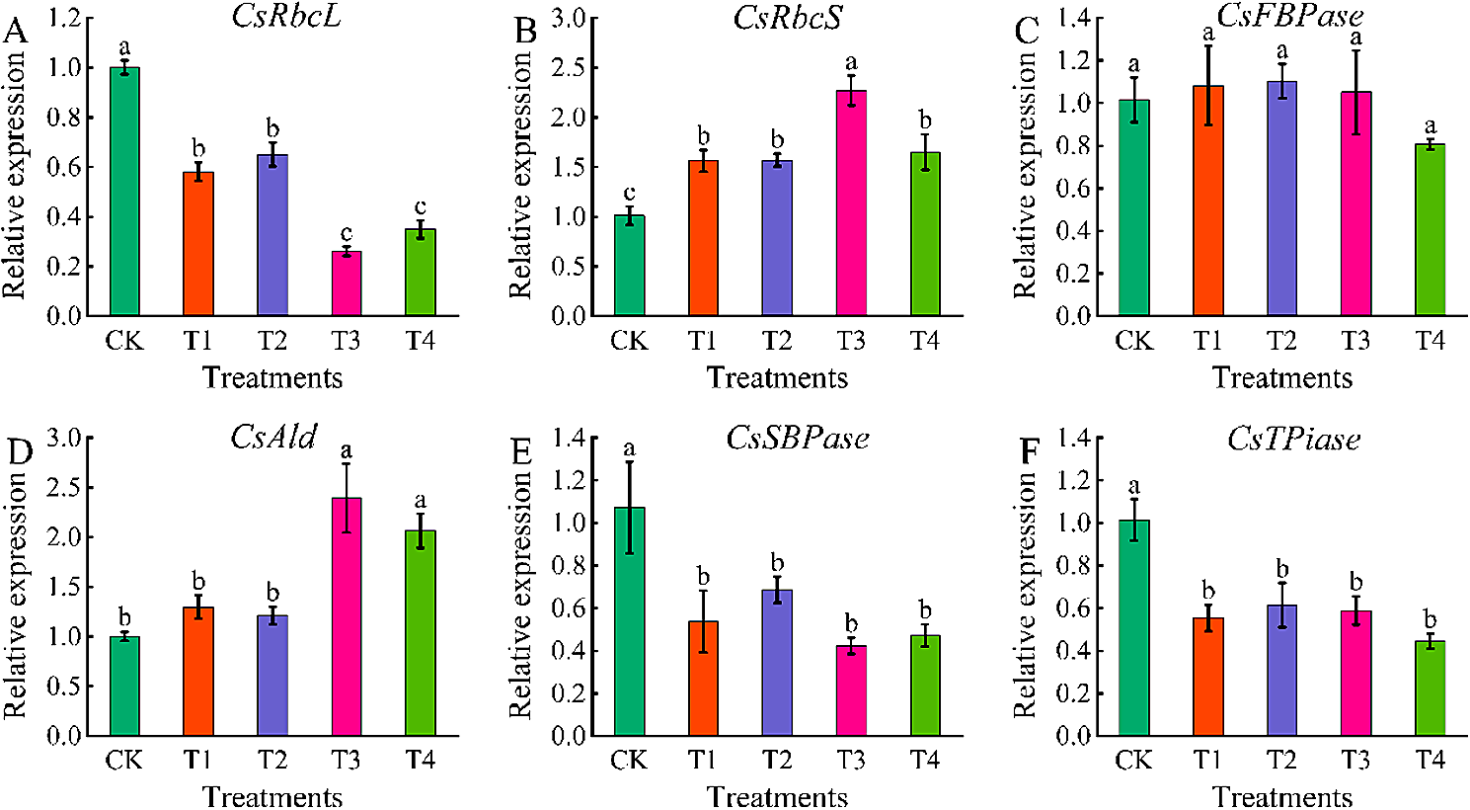
Effects of different concentrations of exogenous EBR on the expression levels of key carbon assimilation enzyme genes in coriander leaves. (A): *CsRbcL* (B): *CsRbcLS* (C): *CsFBPase* (D): *CsAld* (E): *CsSBPase* (F): *CsTPiase*. CK: Water spraying; T1: 0.05 mg.L^− 1^ EBR; T2: 0.1 mg.L^− 1^ EBR; T3: 0.5 mg.L^− 1^ EBR; T4: 1.0 mg.L^− 1^ EBR. Values presented are the means ± SE (*n* = 3). Vertical bars indicate the SE of the means. Different lowercase letters indicate significant differences between treatments (Duncan’s test, *P* < 0.05)

To further study the effects of exogenous EBR application on the photosynthetic capacity of coriander, we determined the relative expression in coriander leaves of genes encoding enzymes acting in carbon assimilation. The relative changes in the expression of key genes for carbon assimilation in coriander treated with exogenous EBR for 7 days are shown in Fig. 6. Compared with CK, T3 treatment significantly increased the expression level of *CsRbcS*. T3 and T4 treatments significantly increased the expression level of *CsAld*, and the relative expression levels of *CsRbcL*, *CsSBPase*, and *CsTPiase* were significantly down-regulated in the T1-T4 treatments. There was no significant difference in the expression level of *CsFBPase* in T1-T4 treatments (*P* < 0.05). *CsRbcS*, *CsFBPase* and *CsAld* first increased then decreased with increasing exogenous EBR concentrations. Compared to CK, the relative expression of *CsRbcS* and *CsAld* was up-regulated by 124.02% and 138.49% under T3 treatment, respectively, and *CsAld* under T4 treatment was up-regulated by 105.71%.

### Correlation analysis

**Fig. 7:**
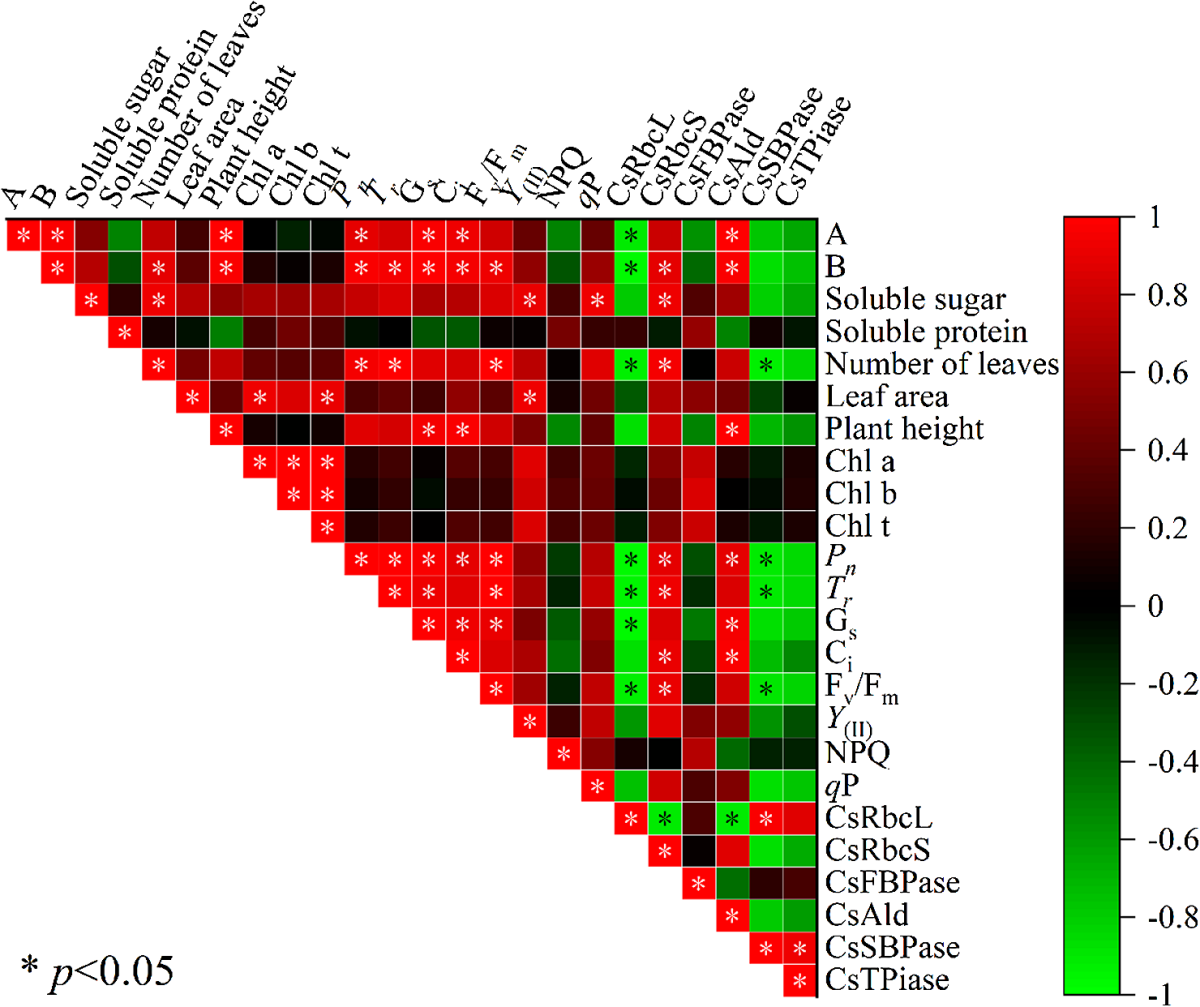
Pearson’s correlation analysis of various indices under different concentrations of exogenous EBR. A: aboveground fresh weight, B: aboveground dry weight

Correlation analysis was performed on the test indicators (Fig. 7). The results of the correlation analysis showed that the aboveground fresh weight under exogenous EBR treatments was significantly positively correlated with the aboveground dry weight, plant height, *P*_*n*_, *G*_*s*_, *C*_*i*_, and *CsAld* (*P* < 0.05), and the aboveground dry weight was significantly positively correlated with the number of leaves, plant height, *P*_*n*_, *T*_*r*_, *G*_*s*_, *C*_*i*_, *F*_*v*_*/F*_*m*_, *CsRbcS*, and *CsAld*. The soluble sugar content was positively correlated with the number of leaves, *Y*_(II)_, *q*P, and *CsRbcS*, plant height was positively correlated with *G*_*s*_, *C*_*i*_, and *CsAld*, *P*_*n*_ was positively correlated with *T*_*r*_, *G*_*s*_, *C*_*i*_, *F*_*v*_*/F*_*m*_, *CsRbcS*, and *CsAld*. Although the soluble protein content with Chl a, Chl b, Chl t, NPQ, and *CsFBPase* showed a positive correlation trend, it did not reach a significant level. The results indicated that plant height, *P*_*n*_, *G*_*s*_, *C*_*i*_, and *CsAld* of coriander under exogenous EBR treatment were related to the increase in yield per plant; the number of leaves, plant height, *P*_*n*_, *T*_*r*_, *G*_*s*_, *C*_*i*_, *F*_*v*_*/F*_*m*_, *CsRbcS*, and *CsAld* were correlated with the increase in aboveground dry weight of coriander; and the number of leaves, *Y*_(II)_, *q*P, and *CsRbcS* were correlated with the increase in the soluble sugar content of coriander.

### Principal component analysis (PCA)

**Fig. 8:**
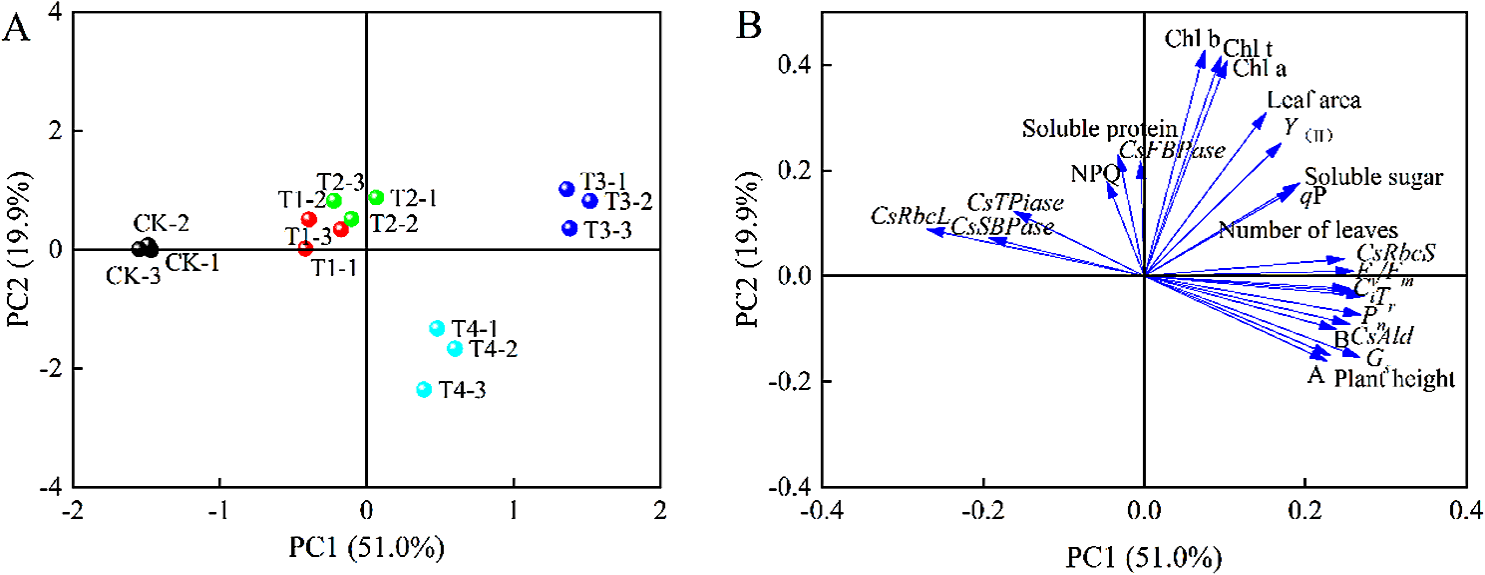
Principal component analysis (PCA), **(A)**: PCA score plot, **(B)**: PCA loading plot. **A**: aboveground fresh weight, **B**: aboveground dry weight. CK: Water spraying; T1: 0.05 mg.L^− 1^ EBR; T2: 0.1 mg.L^− 1^ EBR; T3: 0.5 mg.L^− 1^ EBR; T4: 1.0 mg.L^− 1^ EBR.

Classification models of growth physiology, photosynthetic capacity, and quality of coriander treated with different concentrations of exogenous EBR based on PCA are shown in Fig. 8. The first and second principal components captured most of the variation under exogenous EBR treatment at different concentrations. The two principal components explained 70.9% of the total variance, whereas PC1 and PC2 explained 51% and 19.1% of the total variance, respectively. In the PCA score plot (Fig. 8A), the T1 and T2 treatments were not significantly separated based on PC1 and PC2; however, T1 and T2 were significantly separated from the CK, T3, and T4 treatments, and the CK, T3, and T4 treatments were also significantly separated. Both T1 and T2 were located in the second quadrant, and their effects on the growth physiology, photosynthesis, and quality of coriander were more similar; the T3 treatment was located in the first quadrant, and the T4 treatment was located in the fourth quadrant. In the loading plot (Fig. 8B), Chl a, Chl b, Chl t, leaf area, *Y*_(II)_, *q*P, and soluble sugar were positively loaded onto PC1; yield per plant, plant height, *G*_*s*_, ADMA, and *CsAld* were negatively loaded onto PC2; and NPQ, soluble protein, and *CsFBPase* were positively loaded onto PC2. These indices distinguish the different processing groups of the EBR. *T*_*r*_ and *G*_*s*_ were the main contributors to the first principal component, and Chl a, Chl b, and Chl t were the main contributors to the second principal component. *T*_*r*_, *G*_*s*_, Chl a, Chl b, and Chl t can be used as representative factors to reflect the effects of different EBR concentrations on the growth physiology, photosynthetic capacity, and quality of coriander.

## Discussion

EBR is widely found in plants and exhibits range of biological activities. At suitable concentrations, EBR has been shown to promote crop growth and yield formation [31]. In this study, the effects of 0.05, 0.1, 0.5, and 1 mg.L^− 1^ exogenous EBR on coriander growth and yield were investigated. The results showed that moderate concentration of EBR significantly increased the number of leaves and leaf area of coriander, which is consistent with the results of previous studies on *Brassica juncea L* [32] and cucumber [33]. High EBR concentrations showed a tendency to reduce the number of leaves and leaf area of coriander, which was similar to the results of previous studies on pineapple [34]. The influence of EBR on the leaf length, width, and circumference of coriander was similar to that on the leaf area and number of leaves. Coriander plant height increased significantly under the T3 and T4 treatments, but a trend of decrease in plant height was noted under the T4 treatment relative to theT3 treatment, indicating that the highest concentration of EBR tested here (T4) exceeds the optimum value for plant height. Chen et al. [35] studied the plant height of rice and observed a similar trend.

It has been reported that EBR can improve the yields of peppers [36], cucumbers [37], and peas [38]. The results showed that T3 and T4 significantly increased the aboveground fresh and dry weights of coriander, but T4 showed a decreasing trend compared to T3, indicating that high concentrations of EBR could inhibit the aboveground fresh and dry weights of coriander. Yu et al. [12] showed that exogenous EBR treatment significantly increased the soluble sugar content of cucumbers, whereas Jiang et al. showed that exogenous EBR significantly increased the soluble protein content of cucumbers. Similarly, in this study the optimal concentration of EBR was found to increase the content of soluble sugars and proteins in coriander leaves.

Chlorophyll has a critical role in photosynthesis, and is found in plants, algae, and bacteria. Chlorophyll, is green and absorbs light during photosynthesis. Chlorophyll content reflects the photosynthetic capacity of leaves and the health status of plants [38]. Chl a is the central pigment molecule in the light reaction, Chl b is the light-catching pigment molecule, and the photosynthetic pigment content is closely related to the photosynthetic function of plants. Previous studies have shown that exogenous EBR treatment can improve chlorophyll content in the leaves of various plants, such as wheat [39] and *E. urphylla* [17]. The results of this study showed that exogenous EBR application could increase the contents of Chl a and Chl b in coriander leaves, which is similar to the results of previous studies.

Photosynthesis and transpiration are also regulated by stomata, which are surrounded by pairs of guard cells in the plant epidermis that control gas exchange between the plant and atmosphere [40]. The opening of stomata ensures the exchange of water vapor and CO_2_ inside and outside the plant, providing a channel required for photosynthesis [41]. EBR improves photosynthesis in plants by regulating both stomatal and non-stomatal factors [42]. Appropriate concentrations of EBR have been shown to improve *P*_*n*_, *T*_*r*_, *G*_*s*_, and *C*_*i*_ of tomato leaves [43]and *P*_*n*_, *T*_*r*_, and *G*_*s*_ of *E. urphylla* leaves [44]. The results of this study showed that exogenous EBR treatment significantly increased the *G*_*s*_ and *C*_*i*_ of coriander leaves, indicating that EBR improved the photosynthetic capacity of coriander mainly by influencing stomatal factors. The *P*_*n*_, *T*_*r*_, *G*_*s*_, and *C*_*i*_ of coriander leaves first increased and then decreased as EBR concentrations increased, and reached the highest value under the T3 treatment.

Chlorophyll fluorescence in plants is closely related to photosynthetic efficiency, and analysis of chlorophyll fluorescence kinetics is one of the most powerful and widely used techniques. It can be used as a probe for determining the photosynthetic function of leaves quickly and without damage to the plant [45]. The results of our analyses showed that *F*_*v*_*/F*_*m*_, Y _(II)_, NPQ, and qP of coriander leaves increased to different degrees under exogenous EBR treatments. The *F*_*v*_*/F*_*m*_ of coriander leaf PSII showed a trend of increasing and then decreasing with increasing EBR concentrations. The appropriate concentration of EBR treatment significantly increased the *F*_*v*_*/F*_*m*_ of PSII in coriander leaves, which was similar to the results of previous studies on *E. urophylla* [17]. Some studies have shown that the effect of exogenous EBR on *F*_*v*_*/F*_*m*_ of PSII in peas [46] and cucumbers leaves [15] did not reach a significant level, and the effect of exogenous EBR on the *F*_*v*_*/F*_*m*_ of PSII in leaves may differ depending on the crop species. The trends in variation of *Y*_(II)_, NPQ, and *q*P were similar to those of *F*_*v*_*/F*_*m*_, *Y*_(II)_, and *q*P reached the highest values under the T3 treatment, and NPQ reached the highest values under the T1 treatment, indicating that EBR had a range of concentrations that were suitable for improving the fluorescence parameters of coriander leaves. The increase in NPQ under in the T1 treatment indicates that more light energy captured by chlorophyll may be lost as heat.

The Calvin cycle consists of three stages and a series of complex reactions catalyzed by 11 enzymes. RuBisCO catalyzes CO_2_ assimilation and photorespiration, and determines the net photosynthetic rate of plants [47, 48]. FBPase catalyzes the hydrolysis of fructose-1, 6-diphosphate, and participates in gluconogenesis and Calvin cycle [49]. TPiase and Ald catalyze the reversible conversion of glyceraldehyde-3-phosphate (G3P) to 3-phosphoglycerate (PGA) [50]. SBPase catalyzes the dephosphorylation of sedoheptulose-1,7-bisphosphate, a reaction located at a branching point between the regenerative stage of the Calvin cycle and sucrose or starch biosynthesis, due to its specific location, SBPase controls plant carbon flow [47]. However, the effect of EBR on photosynthesis remains unclear. Yu et al. [12] showed that EBR could improve the initial RuBisCO activity of cucumber but had no significant effect on the RuBisCO content and total RuBisCO activity. In a study by Gao et al. [47] the relative expression of genes encoding dark reaction enzymes synthesis genes was found to be up-regulated 24 h after treatment of maize protoplasts with EBR. These findings are similar to the results of the present study in which *CsRbcS* and *CsFBPase* had high expression under T3 treatment 7 d after the cessation of EBR spraying, indicating that the gene expression levels, protein contents and activities of some dark reaction enzymes did not necessarily increase at the same time under exogenous EBR treatment. This may be the reason why the expression levels of *CsRbcL*, *CsFBPase* and *CsTPiase* were reduced but the net photosynthetic rate was still significantly increased.

## Conclusion

In the present study, exogenous spraying of EBR (0.05, 0.1, 0.5, and 1 mg.L^− 1^) showed varying effects on growth and photosynthetic capacity of coriander. Exogenous EBR treatment increased plant height, promoted leaf growth and aboveground dry weight in coriander, and affected the expression levels of genes encoding carbon assimilating enzymes by increasing leaf chlorophyll content, gas exchange parameters, and chlorophyll fluorescence parameters. Correlation analysis showed that aboveground fresh weight under the exogenous EBR treatment was significantly positively correlated with aboveground dry weight, plant height, *P*_*n*_, *G*_*s*_, *C*_*i*_, and *CsAld* (*P* < 0.05), and soluble sugar content was positively correlated with the number of leaves, *Y*_(II)_, *q*P, and *CsRbcS*. The PCA-based classification model showed a significant separation between the CK control and the treatment groups (T1, T2, T3, and T4), with the T1 and T2 treatments performing similarly. Collectively, the results indicate that exogenous 0.5 mg.L^− 1^ EBR is the best concentration for promoting the growth of coriander, enhancing the photosynthetic capacity of leaves and improving the aboveground fresh weight.

### Electronic supplementary material

Below is the link to the electronic supplementary material.

Supplementary Material 1: RT-qPCR primers design

## Data Availability

All data generated or analyzed during this study are included in this published article.
