# Supplementary material for: Physiological responses of coriander (Coriandrum sativum L.) to exogenous 2,4-epibrassinolide at different concentrations
Source: BMC Plant Biol. 2023 Dec 16;23:649. doi: 10.1186/s12870-023-04684-z (PMC10724981; doi:10.1186/s12870-023-04684-z)
Supplement: Supplementary file 1 — Supplementary Material 1: RT-qPCR primers design [file 12870_2023_4684_MOESM1_ESM.docx]

Supplementary Table 1 RT-qPCR primers design

| Gene name | Gene ID | Forward (5’-3’) | Reverse (5’-3’) |
| --- | --- | --- | --- |
| *CsRbcL* | CsUnG04148 | GGTGTTCTACCCGTTGCTTC | AAGTTCCTCCACCGAACTGT |
| *CsRbcS* | Cs07G02521 | TAGCATGGTGGCTCCTTTCA | CGTTGCTGGTAATGGAGGTG |
| *CsFBPase* | Cs10G01328 | GCTTGCAACAAGACTGAGGT | ACGTGCTAAGTTGGGTCTGA |
| *CsAld* | Cs10G02675 | TCTCCTCAAGCCTAGCATGG | GGACAGCTGGAGGGATTCTT |
| *CsSBPase* | Cs02G01726 | TTGCAGTTGACATGCTTGCT | CCTTCAACTGGACCTCCCAT |
| *CsTPiase* | Cs10G02702 | CTTCACCACAGCAAGCTCAG | CTTCCTCTTTGGCCAGTTCG |
